# Toward biomimetic optogenetics: Drug-free activation of acute opiate reward

**DOI:** 10.1016/j.isci.2025.113465

**Published:** 2025-09-01

**Authors:** Lyla El-Fayomi, Hendrik Steenland, Sabine Lovejoy, Michael Bergamini, Derek van der Kooy

**Affiliations:** 1 Department of Molecular Genetics, University of Toronto, Terrence Donnelly Centre for Cellular & Biomolecular Research, Toronto ON M5S 3E1, Canada; 2 Neurotek Innovative Technology, Incorporated, Toronto ON M6C 3A2, Canada; 3 Institute of Medical Science, University of Toronto, Terrence Donnelly Centre for Cellular & Biomolecular Research, Toronto ON M5S 3E1, Canada

**Keywords:** Natural sciences, Biological sciences, Neuroscience, Systems neuroscience

## Abstract

Functional brain mapping studies typically involve tonic optogenetic stimulation. This does not always reflect natural firing patterns, which could be critical for information encoding. Adopting a biomimetic approach to optogenetics – wherein stimulation intervals mirror those observed *in vivo* to reflect context – we reconcile conflicting data on ventral tegmental area (VTA) GABA neurons in acute opiate reward. Activation using laser pulse sequences mimicking morphine-induced firing patterns is rewarding, while continuous light is aversive. When interspike intervals in rewarding morphine firing patterns are randomized, aversions also result, demonstrating the importance of temporal encoding in this system. We further establish the existence of VTA GABA projections to the tegmental pedunculopontine nucleus that drive reward in a dopamine-independent manner. Overall, our findings are consistent with non-optical studies characterizing VTA GABA neurons, and explain why previous optogenetic manipulations failed to corroborate those outcomes; proof-of-principle that temporal firing patterns are of critical importance in optogenetic toolkits.

## Highlights

• Optogenetic replay of morphine-induced firing in VTA GABA neurons is rewarding
• When rewarding morphine-patterned stimulation files are shuffled, aversions result
• VTA GABA projections to the TPP can drive reward in a dopamine-independent manner
• Temporal firing patterns may be of critical importance in optogenetic toolkits

## Graphical abstract

**Figure:**
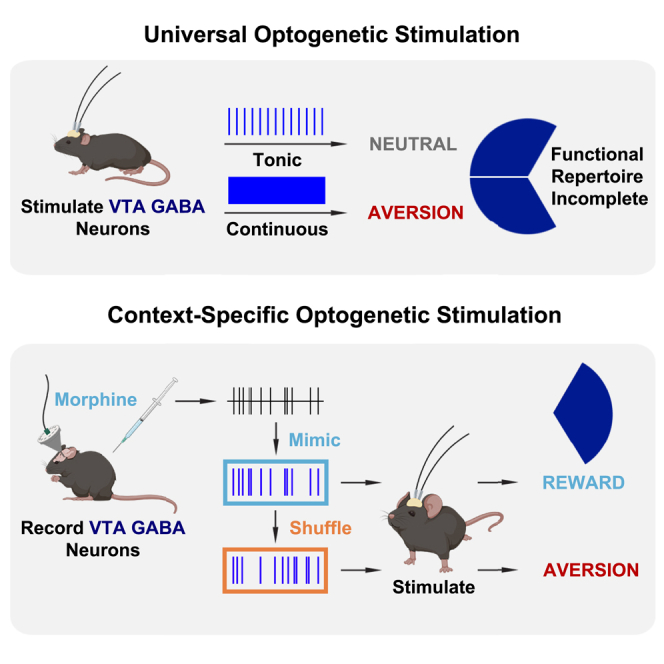

## Introduction

Contemporary functional brain mapping studies have typically involved tonic optogenetic stimulation, wherein light pulses are generated at fixed, regular intervals.^1^^,^^2^ Though this type of stimulation might accurately replicate the activity of neurons that naturally fire tonically, it does not necessarily reflect the vast array of non-tonic firing patterns in the brain.^3^ On the one hand, this broad-spectrum approach is consistent with rate coding theory, which argues that neural information is encoded in average firing frequency.^4^ However, temporal coding theory suggests that this method may result in the loss of any information encoded in individual spike timing and interspike intervals (ISIs), such as timed groups of action potentials (APs).^4^^,^^5^

Given that non-tonic temporal patterns are critical for certain types of information encoding,^2^^,^^5^^,^^6^^,^^7^^,^^8^^,^^9^ it is possible that functional brain mapping using classical tonic activation may not always reveal a) the most physiologically relevant behavior governed by the neurons of interest, b) a complete demonstration of a particular behavior, or c) the full spectrum of behavioral outcomes. In some experiments, tonic stimulation is frequency-matched to the target population’s average firing rate, while in other cases, the stimulation frequency selected depends on the optogenetic tool being used.^1^ Less often are stimulation parameters designed biomimetically, intending to re-construct naturalistic firing patterns and preserve context- or state-specific neural symbols.

We investigated the utility of biomimetically enhanced optogenetics in the context of acute, or naïve, opiate reward processing. The direct involvement of ventral tegmental area (VTA) GABA neurons in driving opiate reward has been hotly debated,^10^ partly owing to data that seem to conflict. Some have cited dopamine (DA) as the primary neurotransmitter involved;^10^ however, this has only been found to be true in opiate-dependent and withdrawn (DW) rodents, as the administration of a D1/D2 receptor antagonist (α-flupenthixol, also referred to as α-flu) abolishes conditioned place preferences (CPP) for morphine in the DW motivational state.^11^^,^^12^

On the other hand, in experiments involving naïve animals that are not in a DW state, the administration of α-flupenthixol at the same dose used previously, or a mutation that depletes DA, does not abolish the rewarding properties of the drug.^11^^,^^12^ This suggests that DA has no bearing on acute opiate reward processing in rodents. In the same vein, VTA infusions of both GABA agonists and antagonists are rewarding through dissociable mechanisms depending on the motivational state of the animal.^13^ In genetic knockouts for the β2 nicotine receptor subunit, selective rescue of the β2 receptor in only VTA GABA neurons restores the acute rewarding effects of nicotine.^14^ These chemical and genetic studies strongly support the involvement of VTA GABA neurons in reward alongside DA, with the key determining factor being the animal’s motivational state.

VTA GABA neurons have prominent projections to the tegmental pedunculopontine nucleus (TPP/PPN). When this site is excitotoxically lesioned, morphine is no longer rewarding in the drug-naïve state.^12^^,^^15^ Overall, these data suggest that VTA GABA neuron projections to the TPP may be critical for the processing of naïve opiate reward.

In recent years, optogenetic studies have yielded results that conflict with non-optical findings; the activation of VTA GABA neurons using continuous light was aversive in conditioned place preference paradigms,^16^ while tonic stimulation at 10–40 Hz produced no significant motivational effect.^17^ To reconcile these bodies of work, we hypothesized that individual VTA GABA neurons could be capable of encoding reward in temporal patterns that are not adequately reconstituted by continuous or tonic optogenetic stimulation.

## Results

### Building biomimetically enhanced toolkits

**Figure 1:**
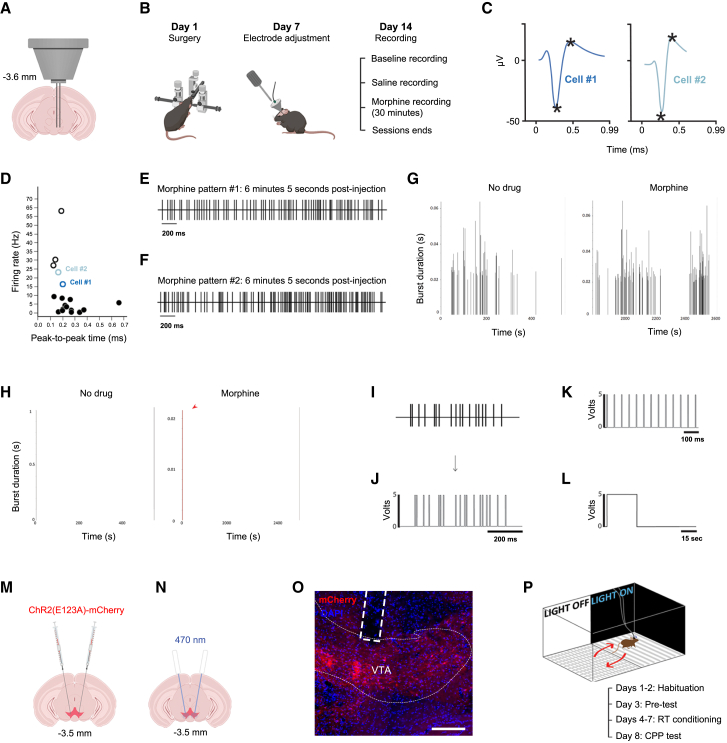
Electrophysiological recordings from VTA GABA neurons under the influence of morphine can be used in real-time place preference paradigms (A) Illustration of an electrode hyperdrive implanted into the VTA. (B) Schedule outlining electrophysiology experiments (*n* = 2 animals).^29^ (C) Waveforms corresponding to selected GABA neurons. Each cell (cell #1 and cell #2) was from a different animal. (D) GABA neurons (black or blue hollow points) fire above 15 Hz (average) with shorter APs relative to DA neurons (solid black). Baseline data is plotted. (E) Representative raster sample from MP #1 (cell #1) 6 min 5 s after morphine injection. (F) Sample from MP #2 (cell #2) at the same time point as E. (G) Plots display detected bursts only. Burst analysis, cell #2. (H) Burst analysis, cell #1. Single burst is observed under morphine conditions (indicated in red). (I) Sample of VTA GABA neuron activity (pictured: 6 min 7 s post-morphine injection). (J) APs were converted to laser pulses using MATLAB. (K) Tonic 20 Hz pulses. (L) Continuous light cycle: 30 s on, 60 s off. (M) rAAV-EF1a-DIO-ChR2(E123A)-mCherry (ChETA_A_-mCherry) was bilaterally injected into the VTA of GAD65-cre mice. (N) Fiber optic implantation above VTA (pictured, 470 nm illumination volume, ∼1 mm^3^).^30^^,^^31^ (O) ChETA_A_-mCherry expression (red) in GAD65+ VTA neurons. Scale bar, 200 μm. (P) Top, illustration of the RT-PP paradigm. Bottom, experimental schedule. See also Figure S1.

To construct biomimetically enhanced stimulation patterns, we recorded the firing patterns of single VTA GABA neurons in mice after the administration of a morphine injection. First, we surgically implanted an electrode hyperdrive – an *in vivo* electrode array that allows for free movement – into the VTA of drug-naïve mice (Figures 1A and 1B). This device allows for the individual adjustment of electrodes to better isolate neuronal units post-implantation (Figure 1B). We recorded cell activity under drug-free conditions, then later after an IP morphine injection at 5 or 15 mg/kg (Figure 1B). Putative VTA GABA neurons were identified based on well-established electrophysiological properties previously corroborated by opto-tagging studies (and which here serve as a ground truth dataset^18^), including average firing frequencies, waveforms, and peak-to-peak times under baseline (drug-free) conditions^16^^,^^19^^,^^20^^,^^21^^,^^22^^,^^23^^,^^24^^,^^25^^,^^26^^,^^27^^,^^28^ (Figures 1C and 1D; Figure S1). Two GABA cell recordings, each from a different mouse, were selected for testing based on isolation quality to ensure single cells – and thus, uncontaminated patterns – were used for experiments (Figures 1C–1F). The first, referred to as cell #1, was from an animal administered 5 mg/kg morphine, while the second, cell #2, was from an animal administered 15 mg/kg. These two doses were tested in order to determine whether the resultant patterns could be differentially rewarding. Furthermore, given the field’s current knowledge gap pertaining to which elements of neural firing actually encode information, and the degree of preservation of these elements from cell to cell (i.e., how much variance is acceptable), population-level analysis would have been premature in this early phase. We instead chose to focus on a proof-of-principle model, demonstrating the encoding capabilities of single neurons rather than generalizing specific patterns to entire populations. We thus limit our conclusions to the activity of single VTA GABA neurons and, therefore, the *potential* of this cell type as opposed to a clear delineation of specific subgroups or proportions.

Both GABA neurons selected were found to exhibit irregular firing across recording conditions, with cell #2 in particular exhibiting temporal instances of higher-frequency activity (Figures 1G and 1H). Classifiable as bursts, these events were observed variably under both morphine and drug-free conditions, and were thus unrelated to opiate administration (Figures 1G and 1H).

Using MATLAB software, we converted the temporal occurrences of APs to binary laser pulse-triggering events. The result was a biomimetically enhanced optogenetic stimulation file wherein laser pulses (3 ms) directly mirrored the position of each AP detected in our recordings (Figures 1I and 1J). These irregular ISIs are visibly different from tonic and continuous stimulation modes (Figures 1K and 1L).

### Stimulation of ventral tegmental area GABA^GAD65+^ neurons using biomimetically enhanced morphine patterns is rewarding

To ensure that neurons could accurately respond to patterns without frequency constraints, we employed the ultrafast ChR2 variant ChETA_A_ (E123A), which accurately follows up to 200 Hz frequencies.^1^^,^^32^ We carried out bilateral stereotaxic injections of the double floxed construct into the VTA of GAD65-cre mice (Gad2^tm2(cre)Zjh^),^33^^,^^34^^,^^35^ followed by bilateral fiber optic implantation (Figures 1M–1O). After three weeks of expression time, animals were optogenetically conditioned using a real-time place preference (RT-PP) paradigm (Figure 1P). New groups of animals were tested with each stimulation mode examined in order to prevent the formation of re-conditioning-related behavioral biases.

**Figure 2:**
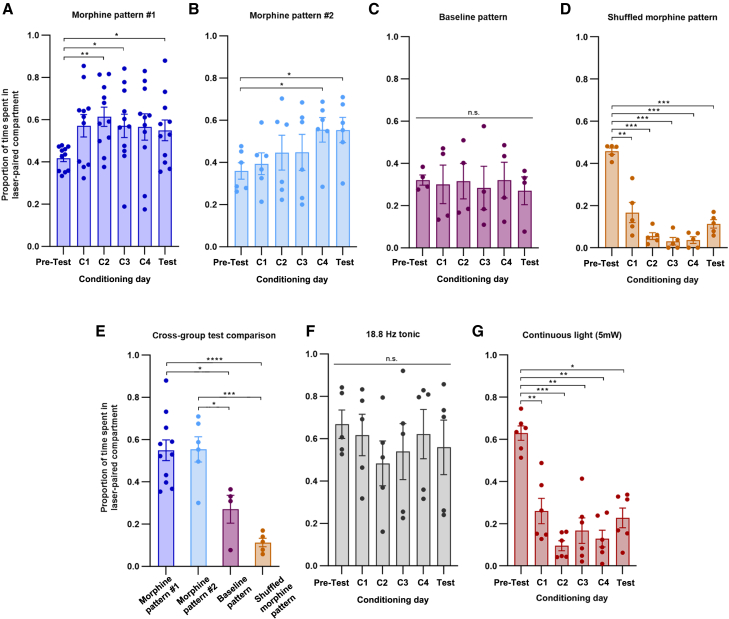
Optogenetic stimulation mirroring morphine-patterned activity is rewarding, while other stimulation modes are not (A) RT preferences for MP #1 (RM ANOVA, *F*_(3.149, 31.49)_ = 3.037, *p* = 0.0414, *n* = 11). Four conditioning days, C1-C4. Scores are significantly higher than pre-test (PT) on C2, C3, and test (T) (Dunnett’s MCT, PT vs. C2: *p* = 0.0012, PT vs. C3: *p* = 0.0452, PT vs. T: *p* = 0.0490). (B) RT preferences for MP #2 (RM ANOVA, *F*_(5,25)_ = 2.8, *p* = 0.0385, *n* = 6). Scores on C4 and T are significantly higher than PT (Dunnett’s MCT, PT vs. C4: *p* = 0.0342, PT vs. T: *p* = 0.0351). (C) BLP has no effect (RM ANOVA, *F*_(2.220, 6.660)_ = 0.1614, *p* = 0.8729, *n* = 4; Dunnett’s MCT n.s.). (D) RT aversions to stimulation using SMP (RM ANOVA, *F*_(1.402, 5.607)_ = 53.35, *p* = 0.0003, *n* = 5). Scores are significantly reduced relative to PT (Dunnett’s MCT, PT vs. C1: *p* = 0.0049, PT vs. C2 & C3: *p* = 0.0003, PT vs. C4: *p* = 0.0002, PT vs. T: *p* = 0.0007). (E) Test scores compared across groups (ANOVA, *F*_(3,22)_ = 14.42, *p* < 0.0001). MP scores were significantly different from BLP (MP #1: Tukey’s MCT, *p* = 0.0126; MP #2: *p* = 0.0234) and SMP (MP #1: *p* < 0.0001; MP #2: *p* = 0.0002) groups. (F) Tonic stimulation (18.8 Hz) produces no effect (RM ANOVA, *F*_(1.608, 6.433)_ = 1.212, *p* = 0.3443, *n* = 5). (G) Continuous stimulation is aversive (RM ANOVA, *F*_(2.019, 10.10)_ = 18.56, *p* = 0.0004, *n* = 6). Scores are significantly reduced across days (Dunnett’s MCT, PT vs. C1: *p* = 0.0014, PT vs. C2: *p* = 0.0004, PT vs. C3: *p* = 0.0093, PT vs. C4: *p* = 0.0021, PT vs. T: *p* = 0.0105). ∗: *p* ≤ 0.05, ∗∗: *p* ≤ 0.01, ∗∗∗: *p* ≤ 0.001. Error bars represent s.e.ms. See also Figure S2.

Mice administered biomimetically enhanced optogenetic stimulation patterned by morphine (morphine pattern, MP, #1 and #2) spent significantly increased amounts of time in the laser-paired compartment across conditioning days and ultimately formed conditioned place preferences upon testing (Figures 2A and 2B). No significant differences in locomotion were detected across trial days (Figure S2). High-frequency burst activity is very unlikely to be the rewarding element, as this type of firing occurs only in MP #2 and not #1, yet both produce the same rewarding effect. Also, there did not appear to be a dose-dependent difference in the degree of reward observed, given that one pattern did not outperform the other (RM two-way ANOVA, treatment/pattern main effect: n.s., *F*_(1, 15)_ = 2.286, *p* = 0.1513; trial day main effect: significant as expected, *F*_(3.171, 47.57)_ = 3.967, *p =* 0.0120; interaction effect: n.s, *F*_(5, 75)_ = 1.545, *p* = 0.1861; post-hoc tests n.s.). This is consistent with the plateau effect observed in the morphine reward literature at doses above 5 mg/kg.^11^^,^^36^ The two patterns thus seem interchangeable.

Taken together, these findings suggest that context-specific firing patterns from single cells hold the potential to unlock behaviors that may not manifest when neurons are stimulated using universal activation strategies, including tonic or continuous light.

### Stimulation patterns shaped by opiate-free contexts are motivationally neutral

We next asked if any non-tonic, temporally irregular stimulation could be rewarding in VTA GABA neurons, rather than features specific to morphine-induced patterning. To test this, we used the drug-free, baseline portion of our recording from cell #1 in the same RT-PP paradigm and found no motivational effect (Baseline pattern, BLP, 16.7 Hz average; Figure 2C). Indeed, test day scores were significantly higher in both MP groups than in the BLP group (Figure 2E), demonstrating that single VTA GABA neurons are capable of encoding and responding to reward signals that are separable from non-reward signals.

### Higher-order interspike interval features are necessary for reward encoding

A critical question regarding the “reward pattern” was whether relative arrangements or repeating sequences of APs could be the main unit of reward encoding. A straightforward way to test this is by examining the interspike intervals. We devised a way to preserve the ISIs themselves while re-arranging their temporal occurrences relative to one another, thereby destroying any repeating sequences: We randomized, or shuffled, the ISIs recorded in MP #1 (Figure S3). When this shuffled morphine pattern (SMP) was used to stimulate VTA GABA neurons, the animals vigorously avoided the paired compartment across days (Figure 2D), indicating that specific arrangements of ISIs may be necessary for the encoding of naïve morphine reward. Again, test day scores were significantly lower in the SMP group as compared to both MP groups (Figure 2E). This suggests that temporally specific activity patterns may be relevant to reward encoding.

### Interrogating rate encoding theory: Average firing frequency is not the key to reward

To determine whether the average firing frequency of a given morphine pattern could be the key to reward, emphasizing rate rather than temporal coding, we generated a frequency-matched tonic stimulation file corresponding to the average firing rate of MP #1 (18.8 Hz) and applied it to the same experimental paradigm. Stimulation produced no detectable change in preference (Figure 2F), suggesting that average frequency is not the factor driving the observed reward responses. These findings are consistent with work by Hughes et al., who stimulated VTA GABA neurons using tonic pulses from 10 to 40 Hz and found no motivational effect. Given that morphine pattern #2’s average frequency falls within this range at 22.2 Hz, a separate experiment to specifically address cell #2 was not required.

### Continuous stimulation of ventral tegmental area GABA neurons is aversive

To demonstrate the full repertoire of VTA GABA-driven behavior, we replicated the findings of Tan et al.^16^ by employing continuous, non-pulsed stimulation in an RT-PP assay. In order to match their experimental conditions as closely as possible, we used the ChR2(H134R) variant in place of ChETA_A_ and set light power to 5 mW at the fiber tip. Matching the opsin variant used previously was especially important in this context, as continuous light stimulation of a channel that can follow up to 40 Hz (with special exceptions, depending on the neuron’s properties) will likely produce different neural firing than a channel that can follow up to 200 Hz, such as ChETA_A._ Thus, applying the ChETA_A_ here might not recapitulate the neural firing mode induced by Tan et al., defeating the purpose of the experiment. This set of trials, which we lengthened to four days, yielded dramatic aversions (Figure 2G). Tan et al. have already ruled out the possibility of light-generated heat driving these behavioral effects at the outlined parameters.

### Reward is encoded along ventral tegmental area GABAergic projections to the tegmental pedunculopontine nucleus

**Figure 3:**
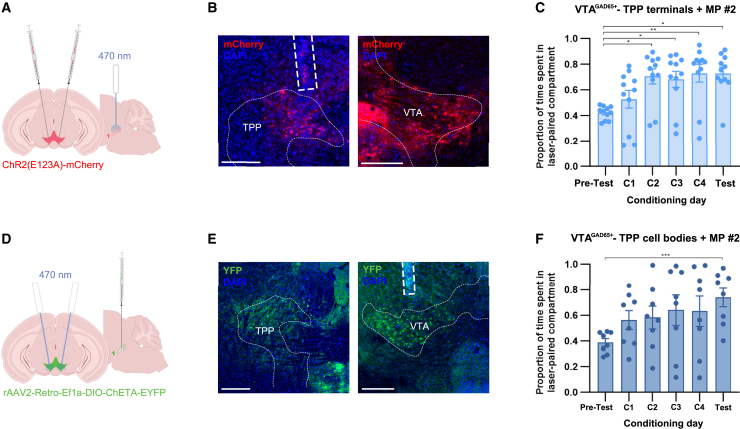
Stimulation of VTA GABAergic projections to the TPP yields robust reward (A) ChETA_A_-mCherry was bilaterally injected into the VTA of GAD65-cre mice. Bilateral fiber optic implantation above the TPP. (B) Left, GAD65+ VTA terminals in the TPP expressing ChETA_A_-mCherry (red). Scale bar, 250 μm. Right, ChETA_A_-mCherry expression in GAD65+ VTA cell bodies. Scale bar, 250 μm. (C) Mean RT preferences for the stimulation of VTA GABAergic terminals in the TPP using MP #2 (Friedman test, *p* = 0.0002, *n* = 11). Scores are significantly elevated relative to PT (Dunn’s MCT, PT vs. C2: *p* = 0.0312, PT vs. C3: *p* = 0.0438, PT vs. C4: *p* = 0.0013, PT vs. T: *p* = 0.0219). (D) rAAV2-Retro-ChETA-EYFP was injected into the TPP. Fiber optics were implanted above the VTA. (E) Left, GAD65+ cells in the TPP expressing ChETA-EYFP (green). Scale bar, 250 μm. Right, GAD65+ VTA cell bodies expressing ChETA-EYFP. Scale bar, 250 μm. (F) Stimulation of TPP-projecting VTA GABA cell bodies with MP #2 yielded reward (RM ANOVA, *F*_(3.024, 21.17)_ = 4.840, *p* = 0.0101, *n* = 8), significant on test day (Dunnett’s MCT, PT vs. T: *p* = 0.0007). ∗: *p* ≤ 0.05, ∗∗: *p* ≤ 0.01, ∗∗∗: *p* ≤ 0.001. Error bars represent s.e.ms.

Given that VTA GABA neurons project to the TPP, and because excitotoxic TPP lesions block CPPs for morphine in drug-naïve animals, we hypothesized that this projection might be critical for naïve reward processing. Moreover, free from the influence of potentially aversive VTA GABA subpopulations that may project elsewhere,^27^^,^^37^ we further hypothesized that the rewarding effects of stimulating this neuron population could be stronger and more consistent than the *en masse* activation of VTA GABA neurons. After again injecting the ChETA_A_ construct into the VTA of GAD65-Cre animals, we placed fiber optic implants over the TPP, in the pons (Figures 3A and 3B). We randomly selected MP #2 for use in this group (given that MP #1 and MP #2 produce the same behavioral effects), then stimulated VTA GABA terminals in the TPP throughout an RT-PP assay. This manipulation resulted in robust reward (Figure 3C). The overall degree of reward was indeed significantly higher than scores observed when VTA GABA cell bodies were targeted with the same pattern, MP #2 (RM two-way ANOVA, treatment main effect: *F*_(1, 15)_ = 5.746, *p* = 0.030; trial day main effect: *F*_(3.317, 49.76)_ = 10.76, *p* < 0.0001; interaction group: n.s. per expectation, *F*_(5, 75)_ = 1.318, *p* = 0.2656; Fisher’s LSD, C2: *p* = 0.0285, T: *p* = 0.0444). Therefore, the activation of this projection is sufficient to elicit reward and produces stronger rewarding effects overall.

Next, to ensure that cell body stimulation of this particular subpopulation of GABA neurons with projections to the TPP could produce similar effects, we injected a custom-ordered monosynaptic retrograde vector,^38^ rAAV2-Retro-Ef1a-DIO-ChETA-YFP, into the TPP of GAD65-Cre mice and implanted fiber optic cables over the VTA (Figures 3D and 3E). This design again captures only the population of GAD65+ GABA neurons that project to the TPP. MP #2 again yielded robust reward by test day (Figure 3F).

### Concurrent activation of aversive ventral tegmental area GABAergic projections to the dorsal raphe may dampen reward during *en masse* cell body stimulation

Given that the GAD65+ VTA GABA neuron population comprises multiple subpopulations with potentially distinctive functions, we sought to investigate whether or not *en masse* activation may simultaneously drive projections that dampen reward by triggering aversive signaling in other regions. One such candidate was an opiate-sensitive subpopulation of GABA neurons located in rostral VTA that projects to the dorsal raphe (DR) and has indeed been found to encode aversion.^37^ This projection is thought to become silenced when morphine is in the system so as not to interfere with reward,^37^ but in our previous optogenetics experiments, it was likely activated in parallel with rewarding projections, potentially interfering with responses. This depends on whether aversions would still be observed if a morphine pattern was used for stimulation, as only 20 Hz pulses have been tested on this terminal field to date.^37^ We thus sought to determine if 1) the firing pattern might dictate different behavioral outcomes (e.g., reward, aversion) in this region of the brain as well, or if 2) aversions would result regardless of morphine patterning since this projection is not usually active during opiate reward.

**Figure 4:**
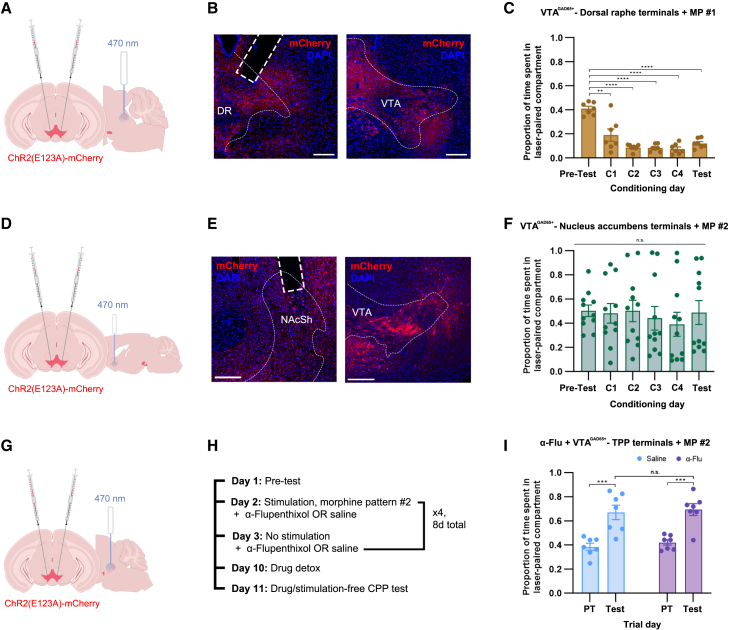
Aversive DR and highly variable NAc effects contrast with dopamine-independent, TPP-projecting reward pathway (A) ChETA_A_-mCherry was bilaterally injected into the VTA of GAD65-cre mice. Fiber optics implanted above the DR. (B) Left, GAD65+ VTA terminals in the DR expressing ChETA_A_-mCherry (red). Scale bar, 200 μm. Right, ChETA_A_-mCherry expression in GAD65+ VTA cell bodies. Scale bar, 200 μm. (C) RT aversions caused by the stimulation of DR terminals using MP #1 (RM ANOVA, *F*_(1.624, 9.744)_ = 37.99, *p* < 0.0001, *n* = 7). Scores are significantly reduced relative to PT (Dunnett’s MCT, PT vs. C1: *p* = 0.0094, PT vs. C2-T: *p* < 0.0001). (D) ChETA_A_-mCherry was bilaterally injected into the VTA of GAD65-cre mice. Fiber optics were implanted above the NAc shell. (E) Top, GAD65+ VTA terminals in the NAcSh expressing ChETA_A_-mCherry. Scale bar, 250 μm. Bottom, ChETA_A_-mCherry expression in GAD65+ VTA cell bodies. Scale bar, 250 μm. (F) Stimulation of NAcSh terminals with MP #2 produces variable results (Friedman test, *p* = 0.6760, *n* = 11). (G) ChETA_A_-mCherry was bilaterally injected into the VTA of GAD65-cre mice. Fiber optics were implanted above the TPP. (H) Experimental outline for CPP paradigm. (I) α-flupenthixol pre-treatment does not interfere with MP #2-driven reward. No significant difference between DA antagonist (*n* = 7) and saline (*n* = 7) group scores (RM two-way ANOVA, trial day main effect: *F*_(1, 12)_ = 64.12, *p* < 0.0001; interaction and injection group n.s.). T scores are significantly higher than PT scores across groups (α-flupenthixol, Šídák’s MCT, *p* = 0.0003; saline, Šídák’s MCT, *p* = 0.0002). ∗: *p* ≤ 0.05, ∗∗: *p* ≤ 0.01, ∗∗∗: *p* ≤ 0.001. Error bars represent s.e.ms.

We injected the same ChETA_A_ construct into the VTA of GAD65-Cre mice and implanted fiber optics over the DR to stimulate VTA GABAergic cell terminals in this region (Figures 4A and 4B). Morphine pattern #1 was randomly selected from the two for this task. Vigorous aversions resulted (Figure 4C), demonstrating that this subpopulation of GABA neurons does not respond positively to morphine reward patterns and thus could dampen reward responses when activated simultaneously with the projections to the TPP.

### Variable motivational effects are driven by nucleus accumbens terminals

The nucleus accumbens (NAc) is a well-established VTA projection site that is critical for reward processing.^39^ While this is usually studied in the context of dopaminergic activity, GABAergic cells of the VTA have also been shown to project to the NAc, and more recently, these GABA projections were found to produce either reward or no effect depending on where the axons terminated in the nucleus.^40^ Given that both of these projections are activated in tandem with others when all GAD65+ neurons in the VTA are stimulated, we sought to investigate how these subpopulations might affect behavior during reward pattern playback in the VTA by targeting a broad area of the NAc shell. This contrasts the activation of one NAc subpopulation at a time (as in Al-Hasani et al. 2021), which in our case, might not have provided insight into the net NAc shell contribution to non-specific, whole-VTA targeting independent of subregional effects. NAc-projecting GAD65+ GABA neurons in the VTA are almost entirely separate from the TPP-projecting population (approx. 1% overlap^41^), so concurrent TPP terminal activation was not a concern.

Fiber optics were implanted over the NAc shell (Figures 4D and 4E), and results were highly variable, with no overall significant differences from pre-test scores (Figure 4F). This high degree of variability strongly contrasts the concise reward responses observed when GABAergic terminals in the TPP were stimulated using a morphine pattern, and provides yet another exemplar of a projection that may interfere with TPP-mediated reward signaling during *en masse* activation.

### GABA-mediated reward in the ventral tegmental area is dopamine-independent

Dopaminergic neurons in the VTA have well-established ties to reward.^39^^,^^42^ To explore the possibility that dopamine might be indirectly involved in the observed GABA-mediated reward responses via local connectivity, especially in their optogenetically synchronized state,^43^ we modified our conditioning paradigm to accommodate drug administration by switching from a real-time to a more classical conditioned place preference paradigm, exposing animals to one compartment in the chamber at a time. As above, the ChETA_A_ construct was injected into the VTA, and fiber optics were implanted over the TPP (Figure 4G). MP #2 was again retained for consistency across VTA^GAD65+^-TPP terminal stimulation experiments (although MP #1 and MP #2 produce statistically interchangeable rewarding effects). Following a pre-test, mice were stimulated in the laser-paired compartment after a pre-treatment (60 min prior to placement in the compartment) with either saline or α-flupenthixol using a dose previously shown to block the rewarding effects of morphine in opiate DW animals via D1 and D2 receptors.^12^^,^^42^^,^^44^ The next day, animals were placed in the opposite compartment and did not receive stimulation, but continued to receive either saline or pre-treatment with α-flu. This design alternated for a total of 8 conditioning days. After a 48-hour drug detoxification period, a final test was conducted in the absence of drug or stimulation (Figure 4H). Both saline and α-flu groups formed statistically significant conditioned place preferences for stimulation with MP #2, and no significant differences were detected between test day scores across saline and α-flu groups (Figure 4I). This result ruled out dopamine as a contributor to the observed GABA-mediated reward in the VTA. It should be noted that any extra-dopaminergic effects (i.e., interactions at, or binding with, other neurotransmitters’ canonical receptors)^45^ also had no bearing on the reward behaviors observed.

**Figure 5:**
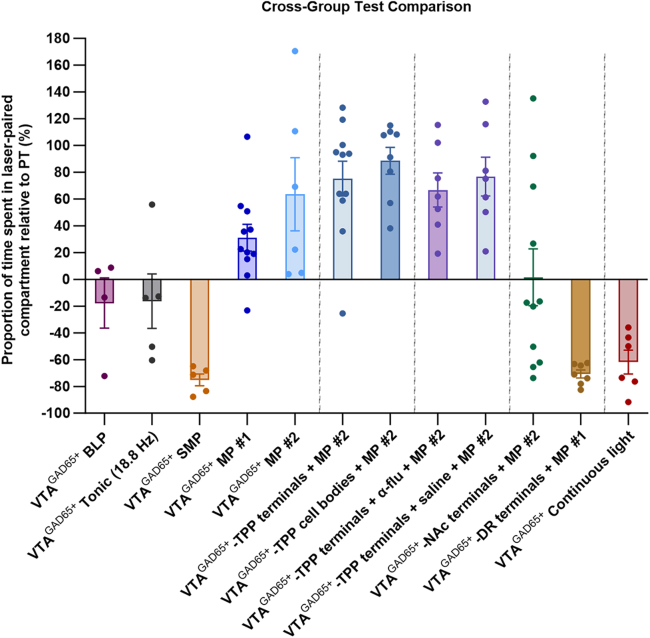
Firing patterns matter: VTA GABA neurons drive a trio of stimulation-specific behavioral responses Mean group scores normalized to percentage change from pre-test, as measured on CPP test day, across experimental conditions. Rewarding effects are observed when VTA GABA neurons are stimulated using morphine patterns; this is true both at the cell body and at terminals within the TPP (with the latter effect being more robust). TPP-mediated reward is dopamine-independent. Aversions are produced at the cell body via continuous light, by shuffling morphine-patterned ISIs, or by stimulating terminals in the DR. No motivational effects are produced when cell body stimulation is tonic, or when baseline patterns are used. Highly variable motivational effects are observed when terminals in the nucleus accumbens are stimulated, potentially indicative of multiple, functionally disparate subpopulations projecting to this site. Error bars represent s.e.ms. Dotted lines separate experiment sets.

Taken together, these data suggest that systemic morphine administered to a drug-naïve mouse produced non-tonic, non-bursting, irregular firing patterns that encode reward in select VTA GABA neurons. This reward is very likely processed via projections to the TPP and is independent of dopaminergic neurotransmission. We also demonstrated that a single VTA GABA cell type can drive a trio of behaviors (reward, aversion, or neutrality) depending on the firing mode and, of course, the projection site (Figure 5). Therefore, firing patterns may be critical for information encoding in certain neuronal populations.

## Discussion

Herein, we show that biomimetically enhanced optogenetic stimulation parameters hold the potential to reveal behaviors that might otherwise go undetected, using VTA GABA neurons as a proof-of-principle. By engineering stimulation patterns that mimic single-neuron activity under the influence of acute morphine, we provide a resolution for a significant data conflict concerning the involvement of VTA GABA neurons in naïve opiate reward behavior.

Temporal coding has long been acknowledged as both valid and important,^6^^,^^7^^,^^8^^,^^9^ despite a lack of widespread theoretical adoption in functional brain mapping studies.^2^ There have, however, been other instances where biomimetic patterns were effectively applied to unlock behavior. For example, VTA DA neurons fire tonically at baseline, but burst in some reinforcement contexts.^46^^,^^47^^,^^48^ These burst parameters were recreated optogenetically and successfully elicited DA-dependent reward behaviors.^49^^,^^50^ Another simple but elegant example is upstream motor control in Parkinson’s disease, wherein optogenetic stimulation using either theta bursts or a single-cell-derived physiological reaching pattern recorded from the motor thalamus was significantly more effective at ameliorating reach-to-grip task performance (a readout of Parkinsonian akinesia) than tonic stimulation.^51^

Our optogenetic findings of reward are consistent with previous, often overlooked literature on this cell type^52^ and elucidate details regarding the nature of information encoding strategies employed by select VTA GABA neurons. When ISIs are re-arranged to yield an artificial activity pattern, reward data is not only lost but replaced by an aversive code. A similar response is seen with continuous light stimulation, which produces a stream of high-frequency spiking at approximately 60 Hz on average.^53^ Given that VTA GABA neurons have been found to fire at around 60 Hz in response to foot shock,^16^ firing induced by continuous light may recapitulate naturalistic aversive signaling. It is important to note that this effect has been found to be unrelated to tissue heating, as continuous light over the VTA of opsin negative mice produced no effect.^16^ The shuffled morphine pattern could also produce the same overall physiological effect as the 60 Hz firing without being perfectly identical, perhaps due to the absence of key reward or neutrality patterns. The result might be a negative punishment effect (wherein something positive is taken away, producing aversions). On the other hand, there may be yet unidentified shared features of each stimulation mode that explicitly encode aversion. One could additionally speculate that an alternative version of the shuffled morphine pattern with a different spike arrangement could either similarly produce the same aversive behavior or drive a different one. Regardless, the findings remain: shuffling the ISIs resulted in the loss of reward, highlighting the importance of a specific temporal code.

In the literature, tonic stimulation was not found to elicit aversions, but was instead motivationally neutral at frequencies between 10 and 40 Hz,^17^ consistent with the results from our tonic, 18.8 Hz stimulation experiment. Tonic stimulation within this frequency range is physiologically relevant to behavior, but in and of itself possesses no motivational valence: it has been shown to drive subpopulations of VTA GABA neurons responsible for head movements on all three axes.^17^ Functionally speaking, it would be detrimental to the animal if tonic firing patterns encoded aversion or reward, as this would make head movements either consistently perturbing or aberrantly rewarding. Of course, the tonic stimulation of specific VTA GABA projections can produce motivated behavior, a notable example being terminals in the ventral pallidum.^54^ Thus, this neutrality effect of tonic stimulation is currently specific to population-level approaches that do not examine one functional subpopulation at a time. However, it is highly plausible that distinct projections can also drive behavior differently or more effectively when patterns are used for stimulation, so further investigation here is merited.

Overall, these data fit into a previously proposed pre-synaptic model of opiate action in the VTA, wherein opiates bind to inhibitory nucleus accumbens terminals synapsing onto VTA GABA neurons,^55^^,^^56^ resulting in a net disinhibitory effect. In this previously morphine-naïve state, GABA projections to the TPP mediate reward, as excitotoxic lesions to this region abolish naïve, but not dependent and withdrawn, opiate reward behavior.^12^^,^^15^ This is supported by our findings, wherein the stimulation of VTA GABA terminals in the TPP produces robust reward, unhindered by other projections that may interfere when cell body stimulation is undertaken (such as mixed messaging from NAc projections and aversive signals from DR terminals).

This proposed motivational model co-exists harmoniously with a role for DA in opiate reinforcement: A BDNF-dependent molecular switch^57^ alters rewarding circuits in states of dependent withdrawal, and morphine then acts in such a way that alters or reduces local GABA release onto DA neurons, allowing for DA signaling in the NAc.^52^ However, as demonstrated by this work and previous studies, dopamine is not required for GABA-mediated reward.^11^^,^^12^

An important theoretical question remains: what advantage might temporal patterning offer over rate coding? Temporal coding was proposed to explain fast and efficient neurobehavioral responses, using precisely timed spikes to communicate specific information at the single-neuron and population level.^58^ This has been suggested to contrast with rate coding, which may be limited by slower information conveyance and longer processing times.^58^ Another feature of temporally coded patterns is the balance achieved by periods of higher activity interspersed with periods of lower activity. Such an arrangement might allow more downstream information through at the projection site relative to a tonic signal, especially in the case of GABA neurons, since they act to inhibit their targets. Currently, there is no unequivocal answer to this question, but the clear importance of temporal patterns has nonetheless been demonstrated in the case of VTA GABA reward encoding.

In terms of the temporal code itself, an important future line of inquiry will be to determine how many neural symbols, or types of activity patterns, encode reward.^4^ Can one cell possess multiple neural symbols encoding the same information? Can symbols differ across cells and still be interpreted similarly by the downstream target? Neuronal *paraphrasing* could be a fascinating possibility. Of special interest will be what qualifies as a true distinction between two codes, meaning how precise the symbol is expected to be and how much spike variation is acceptable before two symbols are considered to be different.^4^ The symbol could even be a rhythm, as this is a common information encoding state in the brain. Once the candidate codes are identified, operant paradigms such as a bar press or nose poke assay can be devised to test them (although the choice of which compartment to enter during an RT-PP assay does have an operant element to it, as compartment entry initiates stimulation). In addition, it will be useful to determine how many GABA neurons in the population partake in this conversation. Again, it is not currently known how representative these recordings are of the entire GABA or reward-encoding subpopulation, and it is not unreasonable to imagine that some VTA GABA neurons could be simultaneously encoding aversion when morphine is in the system due to, for instance, gastrointestinal side effects.

One caveat in efforts to achieve true biomimicry in optogenetics is the stimulation-induced synchrony of all labeled cells. While it is true that the high degree of synchrony induced during optogenetic stimulation may not be physiological and is currently a technological limitation, the current data rule out the possibility that synchrony alone is sufficient for reward. Every stimulation mode we tested induced synchrony, but not all induced reward responses. We thus propose that the biomimicry of even two known firing features – that is activity pattern and, by extension, a physiologically/contextually relevant frequency range – may still unlock previously obscured behaviors despite artificially induced synchrony, and could bring investigators closer to mapping true brain function.

Last, when playing custom patterns back to neurons optogenetically, there will inevitably be baseline activity that occurs in the background, generated by a combination of other neuronal inputs and intrinsic cellular activity. In our case, any neuronal activity that may have been co-occurring with optogenetic playback was insufficient to hinder or prevent reward behaviors from manifesting. It is possible that background activity is captured on the morphine-patterned recordings as well, and so the background activity of the cell targeted for stimulation may simply blend in with that which has been re-constituted optogenetically. It also could be that target neurons filter out baseline activity, ignoring it in the context of a stronger signal, such as opiate-driven reward. The present results demonstrate that native firing patterns may be a critical consideration when using brain stimulation techniques in functional brain mapping studies.

### Limitations of the study

We cannot know and do not claim that every VTA GABA neuron participates in this encoding phenomenon. It is far more likely that there are multiple subpopulations with distinctive functions, especially given what we know about the highly heterogeneous connectivity in the area.^27^

It is true that we also do not know where our recorded cells projected to – the terminal stimulation experiments were merely meant to show that VTA GABA neurons do project to areas that encode morphine-related reward, just as they project to areas encoding aversion, providing a more complete and physiologically relevant view of their functional repertoire.

Another interesting element is whether or not GABAergic projections to the TPP have opioid receptors on their terminals, a question not directly examined here. However, it is known that the TPP is necessary for opiate reward because lesions to this region abolish reward in naïve animals (see ref. 12 and 15, amongst others cited). Furthermore, GABA-B and glutamate synapses in the TPP mediate systemic morphine reward,^59^ carving out an important role for GABA in TPP-mediated naïve morphine reward. It is thus unlikely that VTA GABA neurons would not produce chemical output there, being a major GABAergic input to the region capable of driving reward. However, we acknowledge that there remains a small margin of possibility. Future work should examine this question more closely, as our study had more of a generally technical, optogenetics-focused mandate.

Our initial hypothesis was that tonic stimulation would be aversive, similarly to continuous light, as we had no precedent establishing this mode as neutral at the time. Thus, we paired stimulation to the preferred chamber. While no change in preference was detected across conditioning days, the high initial preferences for the paired chamber at pre-test made it so that test day scores for the same chamber were also high – that is why the interpretation of this result must be carefully taken in context. Pairing a stimulus to the preferred chamber is a risk given that this approach may complicate the detection of preferences; however, given the average trend, the spread of the data, the resultant statistical analysis, plus the data of Hughes et al. 2019, it is far more likely that the correct interpretation of the data in this experiment is neutrality.

Last, even if the cells just beneath the fiber tip are able to reproduce the firing patterns with perfect fidelity, the farther away a neuron is from the light source, the lower the success rate becomes. This means that one will never really achieve 100% fidelity across an entire 3D region. It is also not known whether the downstream target neuron uses every single spike in an incoming message, as suggested above. Thus, in the context of this study, perfect fidelity is less important than whether or not the changes to one’s stimulation mode are sufficient to produce corresponding changes in behavior, and in our case, this has been established. Future work will tease out the specific, critical spike elements important to the behavioral code.

## Resource availability

### Lead contact

Requests for further information, data, or resources should be directed to the lead contact, Dr. Lyla El-Fayomi (lyla.el.fayomi@mail.utoronto.ca).

### Materials availability

The material transfer agreement for the optogenetic constructs was granted by Dr. Karl Deisseroth at Stanford University. This study did not generate any unique reagents.

### Data and code availability

• Data reported in this article will be shared by the lead contact upon request.
• All original code has been deposited at Mendeley Data and is publicly available at https://doi.org/10.17632/ysbgx5y95d.1 as of the date of publication.
• Any additional information required to reanalyze the data reported in this article is available from the lead contact upon request.

## Acknowledgment

The authors thank Brenda Takabe for her training contributions and Taryn Grieder for her suggestions. We also thank the Division of Comparative Medicine at the University of Toronto. This work was supported by a Canadian Institutes of Health Research Foundation Grant to D.V.D.K. (FDN-148407). Elements of the graphical abstract were created in BioRender. El-Fayomi, L. (2025) https://BioRender.com/aw9hpzl.

## Author contributions

L.E. and D.V.D.K. conceived the study. L.E., S.L., and D.V.D.K. designed the experiments. L.E., H.S., S.L., and M.B. conducted the behavioral experiments. L.E. and S.L. completed the histology. L.E. wrote the original draft and generated the figures. L.E., S.L., H.S., and D.V.D.K. reviewed and edited the article.

## Declaration of interests

The authors declare no competing interests.

## STAR★Methods

### Key resources table

**Table:** 

REAGENT or RESOURCE	SOURCE	IDENTIFIER
**Bacterial and virus strains**		

AAV-EF1a-DIO-ChR2(E123A)-mCherry	University of North Carolina Vector Core	NA
AAV-EF1a-DIO-hChR2(H134R)-EYFP	University of North Carolina Vector Core	NA
rAAV2-Retro/Ef1a-DIO-ChETA-EYFP	University of North Carolina Vector Core	NA

**Experimental models: Organisms/strains**		

*Gad2*^*tm2(cre)Zjh*^/J (GAD2-IRES-Cre)	Jackson Laboratory	Stock no.: #010802, RRID: IMSR_JAX:010802

**Software and algorithms**		

MATLAB (R2018B)	MathWorks	RRID: SCR_001622
Spike2 Software	Cambridge Electronic Design	RRID: SCR_000903
MED-PC Software	Med Associates	NA
NeuroExplorer 5 Software	Nex Technologies	RRID: SCR_001818
GraphPad Prism Software	GraphPad Software	RRID: SCR_002798
Original MATLAB Code	This paper	Mendeley Data: https://doi.org/10.17632/ysbgx5y95d.1

### Experimental model and study participant details

#### Mice

All animal use procedures were approved by the University of Toronto Animal Care Committee, in accordance with the guidelines of the Canadian Council on Animal Care. The following mouse line was used: *Gad2*^*tm2(cre)Zjh*^/J (*GAD2-IRES-Cre*, a gift from Z. Josh Huang, Jackson Laboratory stock no. #010802, RRID:IMSR_JAX:010802).^33^^,^^34^ All mice were specific-pathogen-free and were housed on a 12-hour light-dark cycle in a temperature-controlled room. Food and water were provided *ad libitum*. Behavioral studies were completed during the same circadian window, 7:00-19:00. Mice were a minimum of 11 weeks old prior to surgery. Both males and females were used in the study, and sex was not found to influence our results.

### Method details

#### Stereotaxic surgery for *in vivo* electrophysiology

Electrode hyperdrive (8 recording electrodes, HypD-8T-M-LB-NT, Neurotek, Ontario, Canada) implantation surgery was performed under 1.5-2% isoflurane anaesthesia. Male mice were secured in a stereotaxic frame with ear bars and were administered 0.25 mL (2 mg/kg) meloxicam subcutaneously. Ophthalmic ointment was applied to the eyes to prevent drying, and a heating pad was used to maintain body temperature. The skin was disinfected with two successive applications of 70% ethanol and betadine. 2% lidocaine HCl and epinephrine (2-4 mg/kg diluted to a 0.5% solution) were injected at the incision site before opening as a haemostatic agent. 0.5 mL saline was also administered subcutaneously to prevent dehydration. 5 small stainless steel screws (0.75 mm diameter) were placed in the skull for secure mounting (SCR-NT, Neurotek Incorporated) and to ground the device (GND-SCR-NT, Neurotek Incorporated). A rectangular craniotomy was created with the central target at (AP: -3.6, ML: +/- 0.6, DV: 4.2). The dura mater was resected, and a stereotaxic arm was used to position the hyperdrive over the target. The hyperdrive was then fixed in place using dental acrylic. Electrodes used were platinum-iridium (PI20030.5A3, Microprobes, 0.5 MOhm), platinum plated to 100 KOhm with gold and platinum-black using a stimulator (WPI Model# A365) and tested with an impedance meter (IMP2A BAK instruments). Mice were permitted to recover for a week, and electrodes were advanced in 25-50 micron increments toward the dorsal aspect of the VTA while monitoring electrophysiological signals. Putative VTA GABAergic neurons were identified by their short peak-to-trough time and their high firing frequency.

#### Electrophysiological recording

After stable cells were identified, recordings were conducted two weeks post-operatively to allow for full recovery prior to experimentation. On the day of the experiment, the hyperdrive of the mouse was connected to a 32-channel headstage (Intan Technologies, RHD32). The signals were routed via a lightweight, 12-channel cable through a motorized commutator (CMTR12-M-Intan-NT, NeuroTek Incorporated). The signals from the commutator were routed to a data acquisition system (Open Ephys) and displayed in real-time on a PC as thresholded and windowed spike data and continuously sampled data. Data were filtered between 600 Hz and 10 KHz and sampled at 30 KHz. The animals were placed in a transparent container with food and water ad libitum during the recording. A 15-minute baseline recording was first collected. Following this, an IP saline injection was administered, and activity was recorded for another 15 minutes. Lastly, an IP injection of either 5 or 15 mg/kg morphine was administered, and the recording proceeded for 30 minutes. Mice were then returned to their home cage. Putative VTA GABA neurons were identified using electrophysiological markers, including peak-to-trough time and average firing frequency.

#### Spike data analysis

Spike data (∗.spike) were imported into either MClust (version 4.4) operating in MATLAB (R2018B) or Spike2 software (CED) and clustered according to different waveform features (e.g. peak amplitude, energy, trough amplitude, PCA). Average neural waveforms for each cluster were evaluated to determine the time between peak and trough. Neurons with peak-to-trough times less than or equal to 0.3 ms and spike rates equal to or above 15 spikes per second during baseline were considered GABAergic VTA neurons. These criteria have long been established in the literature^16^^,^^19^^,^^20^^,^^21^^,^^22^^,^^23^^,^^24^^,^^25^^,^^26^^,^^27^ and are in agreement with recent data from optical tagging experiments in the VTA,^28^ here serving as a ground truth data set.^18^ Firing rates (spikes/s) were calculated for each experimental condition.

#### Optogenetic pattern reconstruction

After neuronal data were sorted and cut, the timestamps of individual neurons were extracted. The neural times were then converted with custom MATLAB code into pulses (3 ms, designed for optimal ChETA_A_ variant response^32^) ranging from 0 to 1V and saved to a wave file. The wave file was then driven via the audio port of a computer (with volume to control voltage) to a trigger system that generated a digital 5V signal (corresponding to each spike time) to drive laser light stimulation. Signals were observed on an oscilloscope to confirm pulse length prior to delivery. Full, 15-minute recordings were used for stimulation, as the critical elements of each temporal pattern have yet to be determined.

#### In-house fabrication of fibre optic implants

Fibre optic implant construction was completed in two phases, separated by 24h.

Day 1: Fibre optic cable (FT200EMT, Thorlabs) was first stripped (Fibre Stripping Tool, Thorlabs) and trimmed to approximately 1.5 cm fragments using a diamond cutter. Fragments were then gripped using a hemostat rubberized at the tips (Plasti Dip) and manually polished on silicone carbide polishing pads of progressively finer grain (KrellTech). 10-20 passes were completed on 30 μm, 9 μm, 3 μm, and 0.3 μm pads, and subsequently inspected to ensure the polished surface was not angled. A drop of freshly mixed epoxy (Precision Fiber Products, Inc.) was then applied to the flat end of a ceramic ferrule (CFLC230-10, Thorlabs) and the unpolished end of the fibre fragment was passed through the epoxy and into the ferrule until 4.5-5 mm of fibre was left exposed on the polished end (3.9 mm to be inserted into the brain to target VTA, and a minimum of 0.6 mm extra to allow for angled insertion into the tissue, distance from dura to skull surface, and any additional measurement error). This process also results in unpolished fibre protruding at the curved end of the ferrule, which is a necessity. This allows for additional polishing of this untreated end the next day, after the epoxy has cured. After wiping away excess epoxy, 24 hours (minimum) were allowed for the curing process, during which half-completed implants were left in a petri dish lined with double-sided tape at room temperature.

Day 2: After the epoxy had cured, the unpolished fibre could then be treated. If more than 1-2 mm was protruding, nail cutters were used to trim away the extra glass until only 1-2 mm remained. At that point, as above, progressively finer polishing pads were used, except at the following benchmarks: 30 μm was used until just about 0.25 mm remained. 9 μm was used *just* until the glass was flush with the ceramic, but not beyond this point, so as not to sand away any of the ceramic along with the glass. ∼20 passes were then completed on 3 μm and 0.3 μm pads. Both polished ends of the fibre were then patted carefully with a fibre cleaning cloth (FCC-7020, Thorlabs) to remove dust, and then tested to ensure transmission efficiency was >80%. Prior to surgery, implants were sterilized with accelerated hydrogen peroxide (PREempt).

#### Stereotaxic injections and fibre optic implantation

Surgeries for optogenetics were performed under 1.5-2% isoflurane. Male and female mice were shaved using precision trimmers (Philips SatinCompact) and then secured in a stereotaxic frame with ear bars. 0.5 mL of sterile Ringer’s lactate solution and 0.5 mL (20mg/kg) meloxicam were administered subcutaneously. Lubricating eye ointment (Systane) was applied to prevent drying.

After making a ∼1 cm incision in the skin, bregma and lambda were identified. Coordinates were then measured for target structures and craniotomies were drilled (Micromotor drill, Stoelting). Using Neuros syringes (Hamilton) and an automated injection pump (World Precision Instruments), one of three viruses was bilaterally injected into the brain at a rate of 100 nl/min, for a total of 500 nl per hemisphere: 1) In most experiments, AAV-EF1a-DIO-ChR2(E123A)-mCherry (University of North Carolina Vector Core) was bilaterally injected into the VTA (AP -3.5 mm, ML ± 1.1 mm, DV -4.2 mm, 10^o^ angle). 2) To replicate the findings of Tan et al.^16^ we injected AAV-EF1a-DIO-hChR2(H134R)-EYFP (University of North Carolina Vector Core) into the VTA. 3) For the retrograde experiment, rAAV2-Retro/Ef1a-DIO-ChETA-EYFP (custom order, University of North Carolina Vector Core) was injected into the TPP (AP: -4.4, ML: ± 1.3, DV: -2.9, 10^o^ angle). Needles were left in place for ten minutes to allow for viral diffusion. Following injections, 3 screws (mounting screws, HRS Scientific) were placed in the skull, and adhesive (Vetbond tissue adhesive, 3M) was added to the junction where the metal meets bone to enhance implant site stability. Ø200 μm fibre optic implants (fabricated in-house; 0.39 NA fibre, ferrules from Thorlabs) were then lowered into injection craniotomies above the VTA (AP -3.5 mm, ML ± 1.1 mm, DV -3.9 mm, 10^o^ angle), anterior TPP (AP: -4.4, ML: ± 1.3, DV: -2.7, 10^o^ angle), DR (−4.75, 1.3, −2.99, 20^o^ angle), or NAc medial shell (AP: +1.1, ML: ± 1.3, DV: -4.2, 10^o^ angle). Cement (Contemporary Ortho-Jet, Lang Dental) was then applied to fix the implants in place. At the end of surgery, topical antibiotic ointment (Bioderm) was spread over the skin to prevent infection, and the mouse was moved to a clean home cage to recover under a heat lamp.

Surgical improvements made during the study: Replacement of bone screws with dental bonding procedure previously published by Berg et al.^35^ Differences were as follows. Prior to drilling craniotomies, the skull was treated with a 37.5% phosphoric acid gel etchant (Kerr). No screws were placed in the skull. Just prior to fibre optic implantation, a bonding agent (Optibond FL, Kerr) was applied to the skull and UV-cured (Maxima RU1200 Curing Light). Following fibre optic implantation, a UV-curable cement (Gradia direct flo, GC America) was layered overtop the bonding and around the implants. Additional Betaderm was used on the animal’s shaven skin in order to protect it from UV light, and this was prior to additional application for infection prevention. Recovery heat lamps were replaced by heating pads.

#### Real-time place preference

At least 3 weeks following surgery, mice were habituated to laser patch cable (Thorlabs) connection for 15 minutes on two consecutive days prior to experimentation. Habituation sessions were conducted in a new, clean cage to ensure a novel environment was introduced while also preventing too many neutral exposures to the conditioning arena. On pre-test day, no laser stimulation was conducted, and mice were allowed to roam the conditioned place preference chamber (Med Associates) freely for 15 minutes. Assignment of stimulation to a compartment depended on the hypothesis; if the stimulation was thought to be rewarding, it was assigned to the least-preferred compartment based on pre-test scores. If the stimulation was predicted to be aversive, we assigned it to the preferred side. If no predictions could be made, an unbiased procedure was used. All designs were counterbalanced. We then conducted 4 days of real-time conditioning; on each day, one 20-minute session took place. In all optogenetics experiments, laser (470 nm, Laserglow) power was adjusted to be ∼10 mW at the fibre tip unless otherwise indicated, ensuring an illumination volume of 1 mm^3^ that met power requirements for the activation of ChETA_A_ across the entire VTA.^30^^,^^31^ The only exception to this was when we replicated work by Tan et al., where we used 5 mW to match their stimulation parameters and opsin. The patterned laser pulse computer files (3 ms pulse width) or the continuous light file (30 s on, 60 s off) were connected to the laser and played back using one of two custom devices: 1) A manual trigger that takes square wave pulses from the audio jack and generates a 5V pulse to trigger the laser when the file is played (user-controlled, animal supervision required throughout conditioning). In this case, data was acquired using Med-PC software (Med Associates). 2) An automatic laser-triggering device that receives instructions from sensor inserts designed to fit inside the Med Associates CPP chamber and detect animal movement (animal supervision during conditioning not required). This device both acquires and stores data on an SD card. In both cases, stimulation was activated upon entry into the pre-paired compartment and paused when the mouse would leave the paired compartment. Full, 15-minute stimulation files were used during each session and were “played” and “paused” upon entry and exit respectively to avoid loss of potentially rewarding pattern elements. A final day of testing with no stimulation was then conducted, wherein conditions were identical to that of pre-test to ascertain conditioned place preferences or aversions.

#### Classic conditioned place preference

We used the classic CPP assay to condition and test animals in the alpha flupenthixol experiment. Protocol was adapted from.^14^ As above, at least 3 weeks following surgery, mice were split into two pre-treatment groups, one slated to receive alpha flupenthixol (0.8 mg/kg, dissolved in saline) and the other, saline. 0.8 mg/kg was previously found to block D1 and D2 receptors,^44^ and has been used to further demonstrate the blockade of morphine reward but only in the doubly dissociated dependent and withdrawn state – not in the morphine-naïve state.^12^^,^^42^ Intraperitoneal injections were administered 60 minutes prior to conditioning in all cases. Either alpha flupenthixol or saline were paired to both compartments, but stimulation was exclusively paired to one. After habituation (as above), all animals were pre-tested drug- and stimulation-free. Based on these scores, stimulation was assigned to the least-preferred side, since stimulation in this experiment (morphine pattern #2) was predicted to be rewarding. The design was counterbalanced. Exposures alternated days, so that if the first conditioning session (15 minutes) was spent in the laser-paired compartment, the next one would be spent in the control compartment. This lasted a total of 8 days, translating to 4 sessions in the stimulation-paired environment and 4 in the control environment. After conditioning was completed, one day was skipped, allowing the alpha flupenthixol to wash out of the animals’ systems (for a total duration of 48h elapsed since last drug injection). The final test (15 minutes) was then conducted, allowing free access to both chamber compartments.

#### Histology

Mice were administered 0.1 ml of sodium pentobarbital and were transcardially perfused with ice-cold saline followed by 4% PFA. The brain was then carefully dissected from the ventral side of the skull to avoid damaging the fibre optic implants and preserve tracts. Brains were post-fixed overnight in 4% PFA before transfer to a 30% sucrose solution. Tissue was sectioned using a cryostat at -23^o^C, then stained using Hoechst to visualize nuclei. Cre-dependent mCherry expression was used to confirm injection success, and fibre optic tracts were visualized to confirm placement coordinates. Animals with misplaced implants were excluded.

#### Burst analysis

Burst analysis was conducted using NeuroExplorer 5 software (Nex Technologies). Default burst parameters were selected to define a burst: a) Maximum 10 ms between APs within a burst, b) minimum 4 APs per burst, c) minimum 10 ms between bursts, and d) minimum burst duration of 20 ms.

### Quantification and statistical analysis

#### Experimental design and statistical analyses

Both electrophysiological recordings were collected from males so that they could be directly compared to one another with fewer confounding variables. Otherwise, all optogenetic behavioral experiments in the study included both male and female animals, with no exceptions.

Animals were randomly assigned to experimental conditions and were only tested under the influence of a single stimulation type. In behavioral experiments, the experimenter could not be blinded to the training condition, given the nature of optogenetic stimulation. Mice that did not meet *a priori* histological criteria (correct fibre optic placement and successful viral infection in the target region) were excluded from analyses.

For each experiment, full and detailed information regarding the statistical tests used, exact sample sizes, and test results can be found in figure legends.

Data were analyzed using Prism (GraphPad Software). We conducted preliminary analyses to screen for both normality and homogeneity of variance. For datasets that met criteria for parametric testing, one-way, two-way, or repeated measures analyses of variance (ANOVA) were used to assess group differences. If non-parametric tests were required, we used the Friedman test.

Fractional degrees of freedom are sometimes reported here, resultant from application of the Geisser-Greenhouse correction for sphericity. Post-hoc tests were used to directly assess group differences and correct for multiple comparisons following ANOVAs where appropriate. The post-hoc test selected depended on the type of comparison; e.g. Dunnett’s MCT is best when comparing data to a control mean, Šídák’s MCT is used when sets of means are selected for comparison, and so forth. All data were presented as mean ± s.e.ms. Statistical significance was assessed at *p* <0.05. In figures, ∗: *p* ≤ 0.05, ∗∗: *p* ≤ 0.01, ∗∗∗: *p* ≤ 0.001.
