# Supplementary material for: Toward biomimetic optogenetics: Drug-free activation of acute opiate reward
Source: iScience. 2025 Sep 1;28(10):113465. doi: 10.1016/j.isci.2025.113465 (PMC12478058; doi:10.1016/j.isci.2025.113465)
Supplement: Document S1. Figures S1–S3 [file mmc1.pdf]

iScience, Volume *28*

Supplemental information

Toward biomimetic optogenetics: Drug-free activation of acute opiate reward

Lyla El-Fayomi, Hendrik Steenland, Sabine Lovejoy, Michael Bergamini, and Derek van der Kooy

Supplemental Information

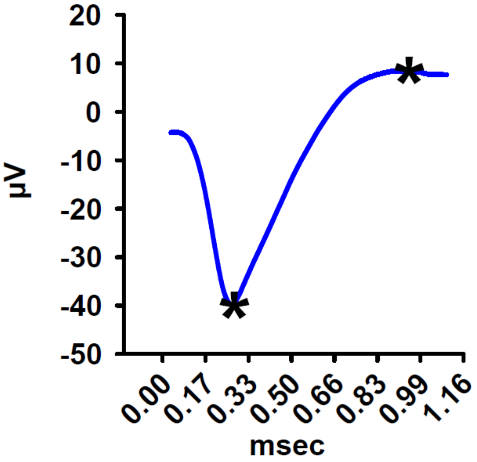

Figure S1. Sample dopamine waveform. Related to Figure 1. Putative dopamine waveform captured from animal 2 during recording. Asterisks indicate the trough and peak of the extracellular spike waveform.

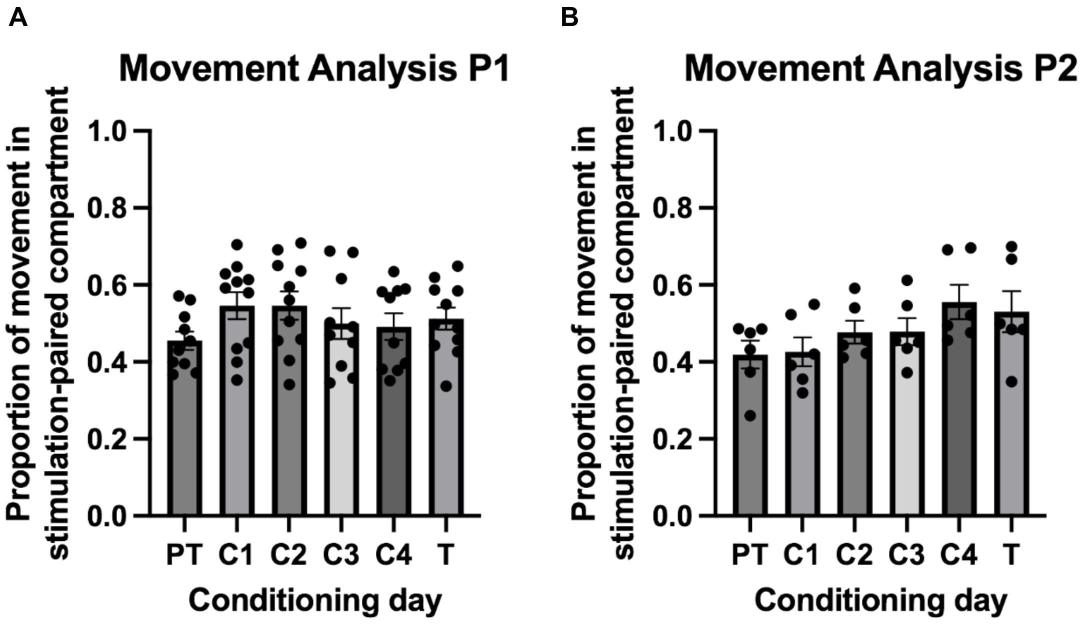

Figure S2. Locomotion in the simulation-paired compartment is not significantly different across days. Related to Figure 2. (A) Proportion of movement in the stimulation-paired compartment (pattern #1) relative to total movement is unaffected across trial days (Mixed-effects analysis, $F_{(2.909, 27.34)}$= 1.548, P=0.2253, n=11; Tukey's MCT n.s.). **(B)** Proportion of movement in the stimulation-paired compartment (pattern #2) relative to total movement is unaffected across trial days (RM ANOVA, $F_{(2.119, 10.60)}$= 2.205,

P=0.1569, n=6; Tukey's MCT n.s.). Error bars represent s.e.ms. In figures, *: $P \leq 0.05$, **: $P \leq 0.01$, ***: $P \leq 0.001$.

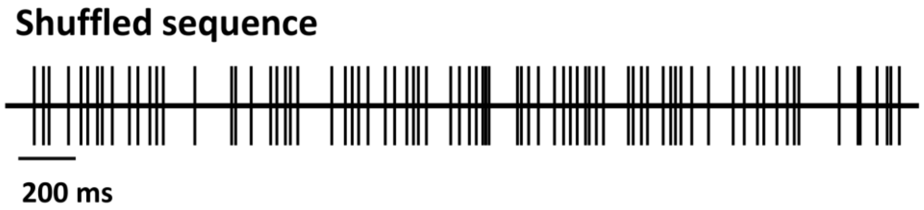

Figure S3. Representative raster sample from the shuffled morphine pattern. Related to Figure 2. Data were sampled 3 seconds into the stimulation file.
